# Qur’anic Views on Human Cloning (I): Doctrinal and Theological Evidences

**DOI:** 10.22074/IJFS.2020.134415

**Published:** 2021-01-27

**Authors:** Mohammad Hossein Nasr-Esfahani, Khadijeh Ahmad-Khanbeigi, Ali Hasannia

**Affiliations:** 1 Department of Animal Biotechnology, Reproductive Biomedicine Research Center, Royan Institute for Biotechnology, ACECR, Isfahan, Iran; 2 Department of Jurisprudence and Law, Research Institute of Imam Khomeini and Islamic Revolution, Tehran, Iran; 3 Department of Qur’an and Hadith Sciences, Shahed University, Tehran, Iran

**Keywords:** Clone, Human Cloning, Theological

## Abstract

**Background:**

Human cloning is a recent occurrence that is not confined to bio-issues; rather, it has provoked numerous questions worldwide and presented scientific and religious challenges. These series of articles aim to examine the proposed approaches and analyze the aspects of human cloning in terms of tenets, morals, jurisprudence, and laws. In this paper, we analyze the ideological and theological evidences, regardless of scientific, ethical and legal problems that exist in the reproduction method.

**Materials and Methods:**

We used a descriptive-analytical method to consider the challenges of human cloning according to the “system of Divine creativity” and “the will of God”, as well as the “pairing system” and “diversity in nature” with emphasis on the Holy Qur’an and Qur’anic commentaries.

**Results:**

According to the Qur’an, although any type of physical changes and retouching of the human body are forbidden, the alteration of God's creation may not prove the prohibition of cloning. Cloning is not contradictory to the principle, precedent, and rule of coupling, and it may be one of the hidden precedents of creation. Therefore, not only does a clone not contradict the precedent of the variety of men, but this variety is a sign for men and not a precedent predominated over the order of nature.

**Conclusion:**

It is proven that cloning does not give life; instead, it utilizes the life bestowed by God. This technique does not contradict the precedents of existence. It is a way to discover some precedents of God and is under the order of cause and effect of the world. Cloning is not considered as a challenge to human beliefs, nor is it a change in Divine creation. Moreover, cloning does not contradict the theological teachings and concepts of the Holy Qur’an and Shiite Muslims.

## Introduction

Once the news of the first mammal (a sheep named Dolly) cloned by a group of Scottish scientists supervised by Ian Wilmut was broadcast (1), the probability of human cloning came into existence (2) and created anxiety among humans. Accordingly, some countries have adopted new laws that pertain to cloning whereas others made changes to their current laws (3). The reaction of monotheists regarding cloning, however, brought serious concerns in many scientific and religious circles. Scholars, on the one hand, have concerns about human cloning and, and on the hand, do not consider it to be a contradiction between science and religion (4-6). Some Shiite scholars, while denying the contradiction of human cloning to Qur’anic verses, state that Almighty God has created man some how, He can reach the secrets of the universe and take possession of them; of course all are inside the casualty rule and the will of God (7). Therefore, if cloning exists in humans, it does not oppose the Unity of God (Tawhid) nor the system of creation (8). However, its legal permission depends on how one uses cloning and the medical syndromes that are to be cured through this technique (9). It must be taken into consideration that the most important principle and source of knowledge about Divine injunctions, as the basis of Islam, is the Holy Qur’an, to the extent that legal and social laws and regulations are rooted in Qur’anic verses and conducts of the Holy Prophet of Islam (pbuh) (8). Therefore, without consideration and research in this area, Muslim scholars cannot legitimize a new area for future study. Numerous verses of the Holy Qur’an call for reflection and ponder, after which Muslims must deeply consider new matters that have not previously existed in order to reach high peaks of new areas. Human cloning consists of different aspects of theology, ethics, and law, each of which may be studied independently. Undoubtedly, this issue is engaged in the fundamental issues of human nature and value, which must be examined through recognizing the angles and various aspects of it. What is considered here is not legal or ethical matters, regardless of their effects. The present research examines and criticizes the most important issues of human cloning in the field of theology, based on the Holy Qur’an and Ahl al-Bayt (pbut).

## Materials and Methods

In this descriptive-analytical study, to reach a reasonable conclusion, our main sources are related to Islamic texts and statements of Muslim scholars. The first and basic source is undoubtedly the Holy Qur’an, the holy book of Muslims revealed from God (10), whose orders and words are accepted by all Muslims. Secondly, the prophetic words (Hadith) and practices (Sunnah), as well as, his family’s (Ahl al-Bayt) statements are considered to be a confirmed source of practicing and issuing fatwas (Islamic legal statements) in Islam. Therefore, by giving general details on human cloning, we divided the Qur’anic views (verses) into four sections. In each section, we discuss and review the statements of the Prophet and Imams (pbut), as well as great Muslim scholars (especially Shi’a) in order to have a sound analysis and respond to the questions of the writing. It is not our intent to prove or reject cloning; the Qur’anic references are studied to reach a common theory between Muslim scholars and provide a possible solution for the present challenges of cloning. We used a descriptive-analytical method with a critical approach in each section. The Results and Conclusion are based on a critical study of the materials.

Among all Islamic documents, only doctrinal and theological evidences related to the issue of cloning were considered instead of the jurisprudential and ethical evidences. Reproductive cloning contains various aspects of studies and each must be reviewed in its appropriate place or position. In this manuscript, apart from all negative aspects of human cloning, if it were to be examined on mankind, we consider it doctrinally and theologically and believe that no scientific discovery is contrary to the eternal power of God, cause of firm beliefs of believers worldwide, or contrary to Muslims’ tenets. Therefore, our aim is an open discussion and forum on this topic by taking into consideration Qur’anic verses and Islamic ideology. Here we discuss the following questions. i. Is cloning an alteration of Divine creation? ii. Is cloning contradictory to the Creatorship of God? iii. Is cloning contradictory to the principle, precedent, and rule of coupling? iv. Is cloning contradictory to the precedent of the variety of men?

## Results

### Literal interpretation of cloning

The definition of cloning is “to make an exact copy of an animal or plant". In English, it is termed "cloning", whereas in French, it is "clonage", and "Istinsakh" in Arabic or copying. Clone is derived from the Greek root "klon" that means a bud and slip of a plant stem used by horticulturists to produce new plants. In other words, clone means the asexual reproduction of a single cell (11).

The similarity between cloning and propagation by a slip of a plant stem is that in both the act of reproduction is carried out in the absence of fecundation. In the first, a slip or root is detached from a tree and planted elsewhere and in the latter, reproduction is also performed in the absence of impregnation.

### Technical aspects of cloning

The asexual reproductive process of a group of living beings that are all genetically the same is called cloning. Briefly, cloning is a series of processes to produce a clone or a copy. Somatic cell nuclear transfer (SCNT) is a technique by which the nuclear substances of a somatic cell are transferred into an enucleated oocyte (the genetic material is removed) to be reprogrammed and initiate development. Finally, an embryo is produced that can be transferred to a surrogate uterus (reproductive cloning) or can be used to produce embryonic stem cells (reproductive cloning) (12, 13). In reproductive cloning, this new living being is a copy of the individual from which the somatic cell was removed (14).

### The goals of human cloning

Today human cloning is divided into two categories: reproductive and therapeutic.

The aim of reproductive cloning is to produce a complete copy of an individual whereas therapeutic cloning aims to produce cells or tissues via stem cell technology for transplantation into an individual with a matched genetic identity. Along with these two applications, cloning has many other implications. Initially, mammalian cloning was performed to answer two fundamental biological questions. Does the nucleus of a well-differentiated somatic cell have all the genetic information for development of an individual? Can a fully differentiated cell be reprogrammed to a totipotent cell? The birth of Dolly provided the answers to these two questions in mammals (15). Subsequently, with the advent of stem cell technology (16), cloning was thought to be the only possible path to produce a patient’s specific stem cells (17) to prevent immune rejection. However, with the advent of induced pluripotent stem cells (IPS) from a well-differentiated cell (18), this aim of cloning subsided and researchers focused on the other use for cloning, which was the production of transgenic animals through SCNT for production of recombinant proteins (19) which were approved by the FDA and other international regulatory agencies. In addition, SCNT has been used for conservation of endangered species in a process called in terspecies SCNT (ISCNT) (20).

### History of cloning

Approximately 100 years prior to the birth of Dolly, a scientist named Jacques Loeb (1859-1924) focused his research on the process of parthenogenesis. Subsequently, Hans Spilman (1983) a German embryologist whose work on the salamander enjoyed universal fame, undertook the first animal cloning experimentations. He succeeded in dividing the embryo of a salamander until the 16^th^ stage. In fact, he demonstrated that the cells reproduced from the first stages of an embryo could once more begin dividing to produce a complete salamander. In 1962, the English biologist John Gurdon (15), cloned a frog by taking the intestinal cells of a tadpole and transferring it to an oocyte whose genetic contents had been removed. Although the extent of success of these experimentations was limited, they positively persuaded researchers to attempt the same procedures on mammals. In 1978, the birth of Louise Brown (21) in England through *in vitro* fertilization opened a new horizon in infertility and experimental biology and paved the way for mammalian cloning (22). On July 5, 1996 at 5pm, Dolly was born. This ewe was the first mammal created through SCNT. However, this event was not announced to the public until February 23, 1997 when Dolly was seven months old. The reputation of Dolly, however, resulted from the impending probability of human cloning. Due to the seriousness of the idea of mammalian cloning, there were various reactions worldwide (23).

### Study of doctrinal and theological evidences of human cloning

### Is cloning an alteration of Divine creation?

The Holy Qur’an (4:118-119), in discussing with polytheists or nonbelievers, mentions Satan or the evil (Shaytan) who claimed that he would mislead people and order them to change creation (10):

"Allah has cursed Satan who said, 'I will surely take of Your servants a settled share, and I will lead them astray and give them [false] hopes, and prompt them to slit the ears of cattle, and I will prompt them to alter Allah's creation.' Whoever takes Satan as a guardian instead of Allah has certainly incurred a manifest loss."

In the Age of Ignorance, the desert settlers of Arabia used to cut their animals' ears in order to forbid people from riding them and these animals became useless. This manner was used to clarify what was unlawful and what was lawful. The above verse was revealed to show this great sin and their heresy (24).

Some consider cloning as one of the examples of this practical change of creatures by Satan. They state that cloning changes human nature and thus breaks the boundaries, by interfering and retouching Divine laws that regulate man’s creation, as well as it is submission to the apparent and hidden devils. They emphasize the phrase: 

"and I will prompt them to alter Allah's creation". Besides this verse, in the verse 30:30, it is forbidden to change creation:

“The nature made by Allah in which He has made men; there it is no altering of Allah's creation, that is the right religion, but most people do not know” (10).

They concluded from these two verses that "altering Allah's creation is forbidden (unlawful or haram)."

These verses are the most fundamental examples for the prohibition of human cloning, which are variously interpreted by most critics. These critics believe that it is unlawful because an alternation of God’s creation occurs with human cloning (25, 26). One jurist who exemplified this verse stated that altering God's creation meant every change or action disproportionate to man's nature [i.e., God prescribed certain tasks for bodily organs and altering them would result in infidelity (kufr)]. Therefore, this is unlawful.

In contrast, some jurists (27, 28) do not rely on these verses; rather, they present many proofs to reject this reasoning and state that it is incorrect to prove prohibition of cloning through these verses.

A contemporary scholar says that cloning in the general sense is not altering Divine creation but it is applying a type of Divine creation by way of discovery which is correspondent with the Divine creation. Thus suggesting that the procedure of cloning or SCNT is not a Divine creation. However, the process is similar to insemination and* in vitro* or* in vivo* fertilization, which results in the development of healthy individuals (27). Similarly, another scholar who rejected this idea has stated that there is no reason to forbid alternation of the creature in general because if it were forbidden, then any type of alternation in nature should be forbidden as well (28).

In a criticism of the above mentioned, it must be stated that, first, a literary review of “khalk-al Allah" (God’s Creation) in the mentioned verse is given followed by remarks from Sunni and Shiite scholars who regard the verses in question.

The author of the commentary Mukatil bin Sulayman interprets khalk-ul Allah (God's creation) as din-ul Allah (religion of God) (29). Moreover, in Lisan-ul ‘Arab for the word khalk by virtue of the verse changing Allah’s creation (falayughayyirunnakhalk-al Allah) it is interpreted as din-ul Allah (changing the religion of God) (30).

Furat al-Kufi (31), in his Tafasir under this verse, narrated from Imam Bakir (pbus): "The connotation of khalk-ul Allah is religion and commands of Allah…". Sheikh Tusi, after quoting different views on this issue, states: "The most compelling explanation of the verse, considering the verse falayughayyirunnakhalk-al Allah (changing Allah’s creation) (verse 30:30), is din-ul Allah (changing the religion of Allah)" (32). Tabrisi comments that alternation of the creature as alternation of the lawful and unlawful of God (33). Bin Kathir quoted from bin ‘Abbas, ‘Akramah and Mujdahid that, by the virtue of the verse 30:30, khalkul Allah means din-ul Allah (34).

Suyuti in his al-Durr al-Manthur reports from bin ‘Abbas that it means religion of God (35). ‘AllamahTabataba’i has stated: "The example of this is the Qur’anic verse 30:30 where Allah states: 'latabdila li khalk-il Allah'. ‘There is no altering Allah's creation,' and then the altering Allah's creation means to come out from the injunction of nature and to leave the right religion"(24). In Tafsir-iNimuni this phrase is interpreted as: "to change the (genius) Divine nature (to exchange the monotheistic nature by polytheism)" (36). In Ruh all-Ma‘ani it is commentated as altering Divine nature, considering the Qur’an: 30:30 (37).

In conclusion, from these points of views of many scholars, this verse and its key words, (falayughayyirunnakhalk-al Allah [to alter Allah’s creation]), which is considered to be cloning by some scholars, is referred to as the alteration of Divine religion and commands and not physical changes. Moreover, it is not mentioned in general to cover all changes with the result of turning many permitted acts (mubah or lawful) into unlawful ones. Breeding plants and animals are common practices throughout history, which could be interpreted as altering God's creation according to this theory. Therefore,*in vitro* fertilization and cloning, which are implications of natural biological mechanisms, are generally considered to be permissible acts by the majority of jurists. In conclusion, according to this verse, any type of physical changes and retouching of the human body are the subjects of this injunction. According to the Qur’anic verses 4:117-119 (10), possibly the direct conclusion drawn from this verse is not correct and an interpretation of one verse should be based on the other verses. Therefore, altering God's creation may not prove the prohibition of cloning.

### Is cloning contradictory to the Creatorship of God?

In many verses the Holy Qur’an attributes creation and existence to God. In verse 7:54, it is explicitly written (10): 

"Look! All creation and command belong to Him. Blessed is Allah, the Lord of all the worlds."

In verse 2:258:

"Have you not regarded him who argued with Abraham about his Lord, because Allah had given him kingdom? When Abraham said, 'My Lord is He Who gives life and brings death,' he replied, 'I [too] give life and bring death.' Abraham said, 'Indeed Allah brings the sun from the east; now you bring it from the west.' There at the faithless one was dumb founded. And Allah does not guide the wrongdoing lot."

Some consider cloning as bringing a new creature into existence. They claim that this procedure leads to the doubt that humans can make alternations in a creature that has been created; thus, he can be a creator. People conceive this to be the process of creating living beings. This doubt has persuaded a group of scholars to use this idea as a reason to prohibit cloning. They regard cloning incompatible to the nature of Islamic beliefs. An ignorant person would imagine that men can create in the way that God creates. This false impression would lead to the weakness of simple-hearted men (38). Rabitat-ul ‘Alam al-Islami, in a letter, refers to this question and declares that cloning is not compatible with human nature and it results in a hesitation (25).

On the other hand, some jurists (39, 24) contradict this theory: "On the basis of this sense, people always retouch Divine work; God, the Almighty, says: 'Allah-u Yatawaffaal-'anfusahinamawtiha', If God brings death, so no one can bring death for anyone! These are contradictory to each other. In fact, the work done by God's servant is different from God's work. The work of a farmer also is interference in God's work. The Holy Qur’an says: '’afara’aytummatahruthun? ’a’antumtazra‘unahu ’am nahn-ulzari‘un?' indeed, it is the Creator, the Almighty God, and not the farmer, that gives it birth or replenishes life. Human knowledge hopes to discover the laws of creation and implement them for his aims. Even after myriads of years, man would not be able to create a being out of the rule of creation."One Muslim scholar declares that cloning is creativeness (takhlik) that can be performed by man but creatorship is only for Allah. Cloning is not "creation of life", as some assume or imagine, but is profiting from a life given by Allah (39)

Another scholar, through an explanation of the meanings of khalk (creation) in the Holy Qur’an and determination of the difference between creating from nothing (as refereed in the Qur’an by “fatir”) and the creation or formation of beings according to the sole role of nature, tried to respond to this misconception as follows: "khalk is applied in Qur’anic verses in three meanings: i. 'kadkhalaktuka min kabluwa lam takushay’an'; 'Certainly I created you before when you were nothing'(verse 19:9). Here khalk means creation from nothing. ii. '’innakhalaknahum min tininlazib'; 'Indeed We created them from a viscous clay' (verse 37:11). In this case it means to create a living being from a lifeless thing. iii. 'thummakana ‘alakatanfakhalakafasawwa; 'Then he became a clinging mass; then He created [him] and proportioned [him]'(76:38). In this case it means alternation from another form and not coming into existence from nothing" (24).

In a criticism of the above mentioned, it must be stated that as human knowledge extends to discover creation, man is unable to create a being outside the rules of creation (or rule of Allah). In fact, cloning is not creating a new being, but innovation by scientists with existing biological material and a role set by Allah Himself. This is also true for creation of cells by aid of computer (40).

Furthermore, scientists do not have knowledge regarding characteristics of the human spirit, of which God says: "They question you concerning the Spirit. Say, 'The Spirit is of the command of my Lord'" (verse 17:85). Scientists are unable to make a living cell, out of nothing, much less create a human being.

The manner of the Holy Qur’an is all affairs in the universe are undertaken solely by God's will and is fulfilled by its chain of causes. If knowledge reaches these causes, it puts in the length and process of creation. Cloning is not a misconception in religion, for every scientific advantage could affect and threaten people's (common) beliefs. Many scholars do not count it as a doubt on religion; instead, they consider it to be an explicit example of the Resurrection, the creation of Eve, and the birth of Jesus (41). In conclusion, since this technology is not out of the cause and effect order of the universe nor out of the will of God (8), cloning is not against Monotheism nor the order of creation.

### Is cloning contradictory to the principle, precedent, and rule of coupling?

The Holy Qur’an, verse 49:13 states:

"Indeed we created you from a male and a female, and made you nations and tribes that you may identify yourselves with one another."

The existence is based on the creation of male and female. "In all things we have created pairs so that you may take admonition" (verse 51:49). The world of beings is based on the pairing pre-resumption and it predominates not only throughout the human world but also in all of the animals and plants. The Holy Qur’an frequently points to this phenomenon. "And [We] create you in pairs" (verse 78:8). "And that is He who created the mates, the male and the female, from a drop of [seminal] fluid when emitted" (verse 53:45-46). The Divine precedent of creating mankind is via marriage between a man and woman. According to some people, human cloning alters and annihilates this precedent. Such individuals state that human cloning is on the basis of satisfaction with one sex without the need for the other sex. Rabitat-ul ‘Alam al-Islami on the strength of verse 49:13 mentions human cloning as a violation of the rule of coupling (25).

A group of scholars believe that throughout the Holy Qur’an, whenever it is written about the creation of man and woman, it is referred to in the usual sexual way where the majority of people come into existence through the joining of sperm and ovum. This does not oppose the fact that a human may be born through other methods, as Jesus who did not have a father (42). It is also said that perhaps these verses speak about the common and usual way of reproduction, but they do not refute other methods. One Muslim thinker has stated that if the precedent of coupling is common and covers the entirety of this material world; thus a human would not be able to break it even if he desires because only the legislative laws can be violated and not the ontological ones. Therefore, cloning is not a violation of the rule of the world of being. If it were so, then humans would have power equal to God's and could break the laws by which God created nature. Moreover, if the creation of Adam and the birth of Jesus are miracles, which is a precedence that has dominated over existence, then human cloning may be one example of a hidden precedence that confirms cloning (43).

In a criticism of the above, it must be referred that ’Ahkamal-Kuran, under verse 49:13 writes: "Allah created people of males and females. If He willed, He would be able to create man without them, as creating Adam and Eve or Jesus, without male. In fact, Allah is able to do all these" (44).

In some commentaries such asal-Balagh (45) and Nimune (36), under the verse we read that creation of people from a male and a female refers to the generation of men that originate from Adam and Eve. The source of all is one and there is no superiority of someone to others. Indeed, piety is the true criterion. Al-Tafsir alMubin declares that this verse is not among the verses of injunctions. It indicates that all men and women benefit from equal rights. It does not indicate making laws and violating them (46). Thus, although it is true that many Qur’anic verses emphasize coupling, no verse indicates that it is infinite to this precedent. The coupling precedent is the common method of nature, but not the absolute law. For example, coupling is contradictory to parthenogenesis (another type of reproduction which is well-observed in nature) and opposite to the creation of Adam, who came from soil, Eve, and Jesus who only had a mother. Despite their unusual creations, they came into this world under the precedents that controlled existence. In one word, cloning may be one of these hidden precedents of creation.

Precedent (sunnah in Arabic) literally means way, method, habit, character, tradition, and nature, and technically the Divine precedents or Sunnahs of God are firm rules that comprise the root and base of the world order dictated by the Wise and Exalted God. These rules must exist for the World Order to not be disarranged. The divine precedent in philosophy is called "the World Order" or "the Causes Rule".

Many verses of the Holy Qur’an emphasize that these precedents are firm and changeless. For instance, in verses 30:62 and 48:23 it says:

"And you may not see any change in the divine precedent."

This content is repeated twice in verse 35:43:

“But no change wilt thou find in Allah's way (of dealing): no turning off wilt thou find in Allah's way (of dealing).”

### Is cloning contradictory to the precedent of the variety of men?

God the Almighty, inverse 30:22, regards the variety of beings in the world as one of His signs.

"Among His signs is the creation of the heavens and the earth, and the difference of your languages and colors. There are indeed signs in that for those who know."

Various verses mention this individuality and count it as a sign of God (16:130; 35:27; and 39: 21).

Many scholars state that cloning requires creating identical copies of man and they consider cloning to be opposed to the law of variety. On this issue, Rabitat-ul ‘Alam alIslami states that variety and distinction are precedents of God in the process of creating man, Who enriches human life through evolution, while cloning deprives the cloned human from distinctive attributes that make him unique among his fellow creatures (25)

Criticizing what was mentioned above, it must be referred that Allamah Tabataba’i under verse 22 of Surah Rum (10) says: "The difference of languages here apparently means difference of words which one is Arabic and the other is Persian, and the difference of colors means difference of races according to colors."

Scholars who study about macrocosm exemplify exact verses. They believe that the world of creation and making with its order is only from the All-Wise God. Zuhayli, also in al-Munir, states that the variety of men regarding their sole origin (one father and one mother) is remarkable (47).

This opposition is not a fair assessment of the fact, for the cloned person is not entirely like the original one. The word "clone" (copy) here is applied indulgently because scientifically the cloned person is different from his origin in three aspects:

i. In SCNT, only the somatic genome is from the original (who gives the nuclear substances) and the other genome that exists in the cytoplasm, including the mitochondria, are from donor cytoplasm or the oocyte.

ii. The wombs in where an original and a clone grow are different.

iii. After birth, we could not limit the personal identity of a person to his genetic identity (8). This fact is stated in Article 3 of the International Declaration of Human Genetic Data:

"Each individual has a characteristic genetic make-up. Nevertheless, a person’s identity should not be reduced to genetic characteristics, since it involves complex educational, environmental and personal factors and emotional, social, spiritual and cultural bonds with others and implies a dimension of freedom” (48).

Also, as explained for the abovementioned verses, this variety is a sign for men and not a precedent that predominates over the order of nature. In parthenogenesis, some living beings reproduce by transferring the same genetic structure. A fairly complete similarity is observed in twins from one egg. Harris (49) names God as the Greatest Artisan Portraitist. Consequently, as we mark the difference among beings as a sign of God, why would clones not be counted as a sign from Him? When an entire code of a human being is in a cell by which he can be reproduced and created, therefore it is a sign of God, which indicates that every cell of a man is also a man.

## Discussion

Through general details on human cloning, the Qur’anic views (verses), statements of the Prophet and Imams (pbut), and the great Shiite Muslim scholars were reviewed and discussed in order to reach reasonable conclusions and have appropriate analyses at hand. Then, by aiming not to prove or reject cloning, we studied Qur’anic references to reach a common theory between Muslim scholars and provide a possible solution for the present challenges of cloning. Apart from all negative aspects of human cloning, we considered cloning from doctrinal and theological aspects and believe that no scientific discovery is contrary to the eternal power of God, cause of firm beliefs of believers worldwide, or contrary to Muslims’ tenets. The following questions were discussed. i. Is cloning an alteration of Divine creation? ii. Is cloning contradictory to the Creatorship of God? iii. Is cloning contradictory to the principle, precedent, and rule of coupling? iv. Is cloning contradictory to the precedent of the variety of men? In each section, after a detailed discussion, some of the main sources and documents were studied and criticized, especially different aspects by the Shite scholars. In the first section, it was proved that* in vitro* fertilization and cloning is generally considered a permissible act by the majority of jurists and according to the Qur’an. Any type of physical changes and retouching of the human body are the subjects of this injunction and altering God’s creation may not prove the prohibition of cloning. In the second section, it was proved that, since this technology is not out of the cause and effect order of the universe nor out of the will of God, it is not against Monotheism nor the order of creation. In the third section, we proved that, not only is cloning not contradictory to the principle, precedent, and rule of coupling, but also it may be one of the hidden precedents of creation. Finally, in the fourth section, it was proved that a clone is not contradictory to the precedent of the variety of men; rather, this variety is a sign for men and not a precedent predominated over the order of nature. The differences among beings is a sign of God; therefore, why are clones not counted as signs from Him? When an entire code of a human being is in a cell by which he can be reproduced and created; therefore, it is a sign of God, which indicates that every cell of a man is also a man.

## Conclusion

The above mentioned reasons are most of the proofs considered in relation to human cloning and its relative doctrinal problems. Our study of the views and comparing them to the Qur’anic contemporary and non-contemporary exegeses of Shiite and Sunnite Muslims enabled us to prove that:

i. Cloning is not giving life but it is utilizing the life bestowed by God, the Almighty.

ii. This technique does not contradict the precedents of existence. It is a way to discover some precedents of God and is under the order of cause and effect of the world.

iii. Since Islam calls mankind for reflecting and thinking, this technique is not considered to be a challenge to human beliefs and it is not a change in Divine creation.

iv. Finally, human cloning is not contradictory to any theological teachings and concepts of the Holy Qur’an and Shiite Muslims.
